# Inhibition of smooth muscle contraction and platelet aggregation by peptide 204–212 of lipocortin 5: an attempt to define some structure requirements

**DOI:** 10.1155/S0962935193000146

**Published:** 1993

**Authors:** K. G. Mugridge, C. Becherucci, L. Parente, M. Perretti

**Affiliations:** 1 Department of Biochemical Pharmacology The William Harvey Research Institute The Medical College of Saint Bartholomew's Hospital Charterhouse Square London EC1M 6BQ UK; 2 Istituto Ricerche Immunobiologiche Siena Via Fiorentina 1 Siena 53100 Italy; 3 SIFI SpA Monterosso Zona Industriale Aci S. Antonio Catania 95020 Italy

## Abstract

Peptide 204–212 of lipocortin (LC) 5 inhibited porcine pancreatic phospholipase A_2_ (PLA_2_) induced rat stomach strip contractions and ADP induced rabbit platelet aggregation in a concentration dependent manner (IC_30_ of 10 μM and 400 μM, respectively). The first two amino acids are not necessary since the eptapeptide 206–212 was equipotent in both assays (IC_30_ of 12.5 μM and 420 μM). Of the two pentapeptides 204–208 and 208–212 only the latter showed inhibitory activity in both models although the potency was much reduced (IC_30_ of 170 μM and 630 μM) compared with that of the parent nonapeptide. Comparison of peptide 204–212 effects with those of its analogues on LC1 and LC2 indicate that lysine 208 and aspartic acid 211 are essential in order to maintain a fully active nonapeptide.


Mediators of Inflammation, 2, 103–107 (1993)

### Inhibition of smooth muscle contraction and platelet aggregation by peptide 204–212 of lipocortin 5: an attempt to define some structure requirements

K.G. Mugridge,* C. Becherucci, L. Parente and M. Perretti[CA,1]

Istituto Ricerche Immunobiologiche Siena, Via Fiorentina 1, 53100 Siena, Italy; [1]Department of Biochemical Pharmacology, The William Harvey Research Institute, The Medical College of Saint Bartholomew's Hospital, Charterhouse Square, London EC1M 6BQ, UK

* *Present Address*: SIFI SpA, Monterosso, Zona Industriale Aci S. Antonio, 95020 Catania, Italy

[CA] Corresponding Author

**PEPTIDE 204–212 of lipocortin (LC) 5 inhibited porcine pancreatic phospholipase $A_2$ ($PLA_2$) induced rat stomach strip contractions and ADP induced rabbit platelet aggregation in a concentration dependent manner ($IC_{30}$ of 10 $\mu$M and 400 $\mu$M, respectively). The first two amino acids are not necessary since the eptapeptide 206–212 was equipotent in both assays ($IC_{30}$ of 12.5 $\mu$M and 420 $\mu$M). Of the two pentapeptides 204–208 and 208–212 only the latter showed inhibitory activity in both models although the potency was much reduced ($IC_{30}$ of 170 $\mu$M and 630 $\mu$M) compared with that of the parent nonapeptide. Comparison of peptide 204–212 effects with those of its analogues on LC1 and LC2 indicate that lysine 208 and aspartic acid 211 are essential in order to maintain a fully active nonapeptide.**

**Key words:** Anti-inflammatory peptides, Lipocortin, Phospholipase $A_2$

### Introduction

The term lipocortin (LC) indicates the members of a family of calcium- and phospholipid-binding proteins, also called annexins, whose physiological role remains to be fully clarified.[1] They have been proposed to mediate part of the anti-inflammatory action of glucocorticoid hormones,[1] and to date clear anti-inflammatory activity has been described for three members of this family, LC1,[2] LC2[3] and LC5.[4]

It has been suggested recently that the anti-inflammatory site of human LC1 may be located in region 247–255 which is strictly homologous to portion 39–47 of rabbit uteroglobin, another steroid-inducible anti-$PLA_2$ protein.[5] The nonapeptides corresponding to these high homology regions showed anti-inflammatory activity, and have been named antiflammins (AFs), with AF-1 (Met-Gln-Met-Lys-Lys-Val-Leu-Asp-Ser) being drawn from uteroglobin and AF-2 (His-Asp-Met-Asn-Lys-Val-Leu-Asp-Leu) from LC1. A subsequent study comparing the amino acid sequences of uteroglobin and human LC5 has also determined a high homology between AF-1 and the region 204–212 of LC5.[6] This nonapeptide inhibits $PLA_2$ dependent processes *in vitro* such as release of prostaglandin $E_2$ from stimulated macrophages or fibroblasts. *In vivo,* the LC5 derived peptide has been demonstrated to be anti-inflammatory reducing the oedema caused by the injection of carrageenin into rat paw.[6] In a recent investigation the pharmacological activity of this nonapeptide has been confirmed in another experimental system, i.e. the release of prostacyclin from aorta rings.[7] In this model the inhibition exerted by peptide 204–212 ($IC_{50}$ around 10 $\mu$g/ml) disappeared when arachidonic acid was added to the incubation medium.[7]

However, few studies have investigated the structural requirements of the AFs which may be responsible for the biological activity of these peptides. One report has shown that AFs shorter than nine amino acids lose their *in vitro* anti-$PLA_2$ activity.[5] Another study has shown the four amino acid core sequence (Lys-Val-Leu-Asp) to demonstrate activity by reducing ADP induced platelet aggregation *in vitro.*[8] More recently it has been shown that replacement of methionine with alanine or norleucine at amino acid position 3 of the AFs can be achieved without loss of activity.[9] This study has also pointed out that leucine and serine are interchangeable on position 9.

In the present work the efficacy of a number of fragments derived from peptide 204–212 of LC5 as well as two peptides from corresponding portions of LC1 and LC2 (hereafter referred to as analogues) have been evaluated in two *in vitro* models: ADP

© 1993 Rapid Communications of Oxford Ltd induced rabbit washed platelet aggregation[8] and $PLA_2$ induced rat stomach strip contractions.[6] Based on the data obtained some structural requirements of peptide 204–212 of LC5 and, possibly, of other AFs are discussed.

### Materials and Methods

*Peptides:* Peptides were purchased from Protein & Peptide Research (Reading, UK). Purity was always more than 95% as assessed by HPLC analysis. The correct amino acid composition and molecular weight were checked by mass spectrometry (all data furnished by the manufacturer). The peptides used throughout this study are reported in Table 1.

*ADP induced aggregation of rabit washed platelets:* Male New Zealand rabbits (2.5–3.0 kg body weight) were bled by the aorta and the blood collected in 3.15% sodium citrate. Washed platelets, obtained as described,[10] were suspended at the concentration of $5 \times 10^7$ in 0.45 ml of Tyrode's buffer containing 3 mg/ml of bovine serum albumin. After warming at 37°C, 50 μl of sample, 10 μl of ADP (1 mM; final concentration $2 \times 10^{-5}$ M) and 5 μl of $CaCl_2$ (100 mM) were added simultaneously and aggregation recorded by using a Chronolog Dual Aggregometer (Chronolog Corporation, Havertown, PA, USA). Data are reported as the percentage of control aggregation measured when 50 μl of buffer were used instead of the sample.

*$PLA_2$ induced rat stomach strip contractions:* Isolated stomach strip preparations were prepared from male Wistar rats (250–300 g body weight) and contractions were elicited by incubation of the tissue with porcine pancreatic $PLA_2$ as described previously.[6] In these experimental conditions incubation with indomethacin (2 μg/ml) reduced by 80% the contractile response evoked by $PLA_2$,[6] suggesting that the greater majority of the contraction recorded was due to arachidonic acid release. Stomach strip preparations were suspended under 2.5 g load in 20 ml organ chambers and bathed with gassed (95% $O_2$; 5% $CO_2$) Krebs' solution maintained at 37°C. Tissue contractions were recorded via isotonic transducers (type 7006, Basile, Comerio, Italy) coupled to a dual channel recorder (type 7070, Basile). Preparations were equilibrated for a minimum of 1 h before starting any experimental procedure. To contract tissue, 4.4 μg/ml of porcine pancreatic $PLA_2$ was added to the bathing solution. This concentration has been reported previously to cause approximately 85% of maximum contraction.[6] In all cases, at least two consecutive contractions of similar magnitude were obtained to this concentration of $PLA_2$ before allowing evaluation of the peptides. Peptides were incubated with the isolated tissue for 30 min, after which the preparations were again challenged with the same concentration of agonist.[6] To standardize the contractions obtained from different tissue preparations, responses are expressed as a percentage of the contraction elicited by $PLA_2$ prior to incubation with the test peptides.

*Materials:* Chemicals were purchased from Sigma Chemical Co. (Milan, Italy) while salts of analytical grade were obtained from Merck (Darmstaad, Germany).

Table 1. Peptides used in this study

| Region | Protein | Amino acid sequence 1 2 3 4 5 6 7 8 9 |
|---|---|---|
| 204–212 | LC5 | Ser-His-Leu-Arg-Lys-Val-Phe-Asp-Lys |
| Fragments | | |
| 206–212 | LC5 | Leu-Arg-Lys-Val-Phe-Asp-Lys |
| 208–212 | LC5 | Lys-Val-Phe-Asp-Lys |
| 204–208 | LC5 | Ser-His-Leu-Arg-Lys |
| Analogues | | |
| 231–239 | LC1 | Pro-Gln-Leu-Arg-Arg-Val-Phe-Gln-Lys |
| 223–231 | LC2 | Pro-His-Leu-Gln-Lys-Val-Phe-Asp-Arg |

LC: lipocortin.

### Results

*Effect of peptide 204-212 of LC5 and its fragments:* Figure 1A shows the inhibition exerted by the various peptides on rat stomach strip contractions elicited by $PLA_2$. The maximal inhibition observed was 60–65%. For peptide 204–212 an $IC_{30}$ of 10 μM could be measured. The seven amino acid fragment 206–212 was similarly active ($IC_{30}$ = 12.5 μM). Of the two pentapeptides only peptide 208–212 retained some inhibitory activity ($IC_{30}$ = 170 μM) while the fragment 204–208 was inactive. A similar pattern of effects was observed on ADP induced aggregation although higher concentrations of peptides were required to obtain a significant inhibition (Fig. 2A). Peptide 206–212 caused almost maximal inhibitory effect, although in terms of $IC_{30}$ (420 μM) it was equiactive with the parent nonapeptide (400 μM). However, in this model both pentapeptides were active with an $IC_{30}$ of 630 μM for peptide 208–212 and of 890 μM for peptide 204–208.

*Effect of the analogues of peptide 204-212 of LC5:* When the region 204–212 of LC5 is analysed on LC1 and LC2, following the alignment described by Pepinsky *et al.*,[11] two other nonapeptides can be identified (reported in Table 1). The analogue of LC2 was as active as peptide 204–212 of LC5, with an $IC_{30}$ of 4 μM in the smooth muscle model (Fig. 1B) and of 420 μM in the platelet aggregation system (Fig. 2B). The analogue of LC1 was less

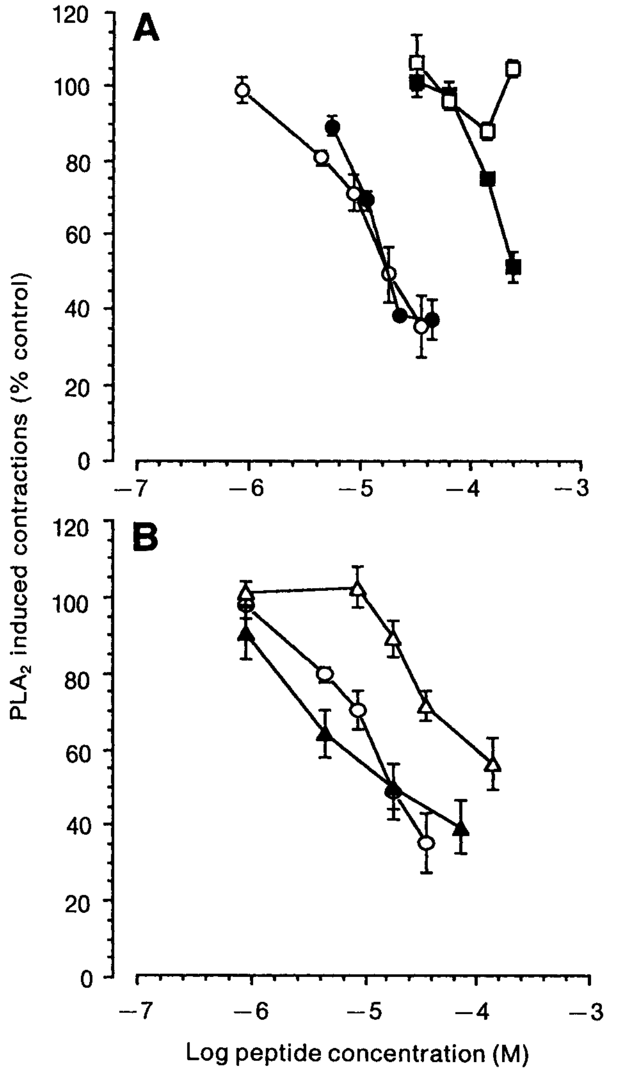

FIG. 1. Effect of peptide 204–212 of lipocortin 5 (LC5) on $PLA_2$ induced rat stomach strip contractions in comparison with the efficacy of its fragments (panel A) and analogues from lipocortin 1 and 2 (LC1, LC2) (panel B). Values are mean ± S.E.M. of 4–9 separate determinations. Values ≤80% of control contractions (induced with $PLA_2$ 4.4 μg/ml) are statistically significant ($p < 0.05$, paired Student's *t* test on original values). Key: ○, 204–212 LC5; ●, 206–212 LC5; ■, 208–212 LC5; □, 204–208 LC5; △, 231–239 LC1; ▲, 223–231 LC2.

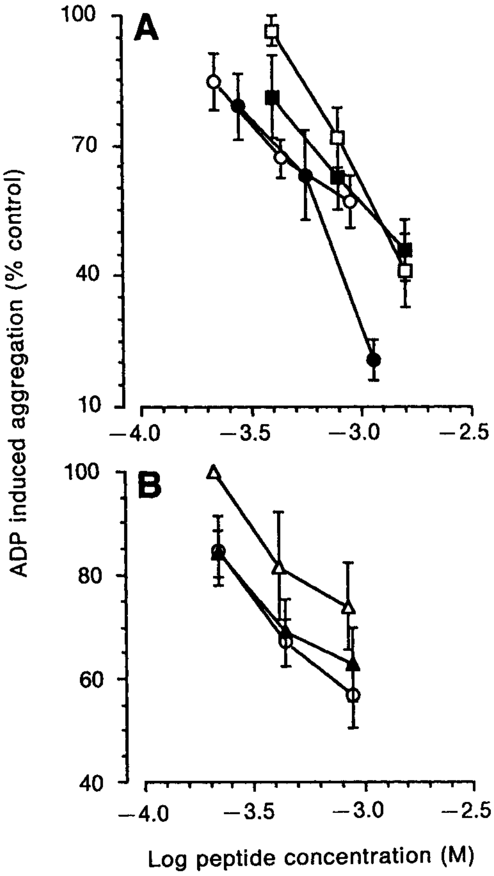

FIG. 2. Effect of peptide 204–212 of lipocortin 5 (LC5) on ADP induced rabbit platelet aggregation in comparison with the activity of its fragments (panel A) and analogues of lipocortin 1 and 2 (LC1, LC2) (panel B). Values are mean ± S.E.M. of 3–10 separate determinations. Values ≤70% of control aggregation (induced by ADP 2 × $10^{-5}$ M) reach the statistical significance ($p < 0.005$, paired Student's *t* test performed on original values). Key as in Fig. 1.

active in both models, with an $IC_{30}$ of 45 μM on stomach strip contractions (Fig. 1B) and an approximate $IC_{30}$ of 1 mM on platelet aggregation (Fig. 2B). In the latter case inhibition of platelet aggregation did not actually reach 30% with any concentration used.

### Discussion

The present study has investigated the biological activity of nonapeptide 204–212 of LC5 and that of related fragments and analogues. For this purpose two *in vitro* experimental models already reported to be affected by AFs, have been used. The first system was the $PLA_2$ elicited rat stomach strip contractions.[6] In this model $PLA_2$ induced contractions are proposed to be due mainly to prostaglandin formation inasmuch as indomethacin exerts profound inhibition (79.1%) of this response.[6] Interestingly, 2 h incubation of stomach strips with dexamethasone resulted in a potent inhibition (62.4%) of the contractions evoked by the stimulus, this effect disappearing in the presence of cycloheximide.[6] This may indicate the involvement of an endogenous LC in the action of the steroid. This observation is substantiated by the fact that human recombinant LC5 inhibits this $PLA_2$ contractile activity (K.G. Mugridge, unpublished data). Peptide 204–212 of LC5 inhibited $PLA_2$ induced contractions in a concentration dependent manner, the effect being selective since it did not affect to any extent the contractile activity elicited by either arachidonic acid or prostaglandin $E_2$.[6] AF-2 was found to be as active as peptide 204–212.[6] The second model used in this study was the ADP induced platelet aggregation which has been reported to be sensitive to both AF-1 and AF-2.[8] Their effectiveness has been related to inhibition of endogenous $PLA_2$ activity, inasmuch as a loss of effect was observed in the presence of arachidonic acid.[8] Similarly, a recent study has shown peptide 204–212 of LC5 to inhibit the spontaneous release of prostacyclin from isolated rat aorta rings but not arachidonic acid-stimulated release.[7]

In both experimental systems, peptide 204–212 exerted a concentration dependent inhibition although different levels of sensitivity were found between the two models. The reason for the lower sensitivity observed in the platelet model is not clear. Washed platelets were used in these experiments to eliminate plasma proteases and therefore the likelihood of an aggressive breakdown of the peptides. However, similar high concentrations of AFs have been used in the same model.[8] The seven amino acid fragment 206–212 fully retained the inhibitory activity of the parent nonapeptide. This eptapeptide was also equipotent to 204–212 in inhibiting the release of prostaglandin $E_2$ from rat peritoneal macrophages stimulated with opsonized zymosan.[6] Taken together, these findings indicate that the first two amino acids of the nonapeptide 204–212 are not necessary to achieve biological activity. The pentapetide 208–212 was active in both systems, albeit with lower potency than the parent nonapeptide. Contrastingly, fragment 204–208 was inactive on $PLA_2$ induced contractions, but exhibited some activity in the less sensitive platelet aggregation model. This effect in the latter model may be related to different mechanism(s). Recently, peptide 204–209 of LC5 has been proposed to represent the sequence responsible for the anticoagulant action, this effect disappearing when histidine 205 is substituted.[12,13]

Compared to AF-2, region 204–212 of the third repeat of LC5 is well conserved in the other members of the LC family, not only in terms of primary structure[11] but also from a three dimensional point of view.[14] The nonapeptide 231–239 of LC1 and the nonapeptide 223–231 of LC2 have more than 50% identity with peptide 204–212 of LC5. They have also a good homology sequence with AF-2. These observations prompted us to evaluate the effect of these analogues. Moreover, taking into account the concept that the first two amino acids of peptide 204–212 are not important to the achievement of the biological activity, the effectiveness of peptide 204–212 and these analogues in relation to the seven amino acid sequence was compared. Indeed, as highlighted in Table 2, peptide 223–231 of LC2 was equiactive to the LC5 derived nonapeptide, this indicating that the fourth and the last amino acids (207 and 212 on peptide 204–212 of LC5) are probably not important. On the contrary, the fragment 231–239 of LC1, differing only in the fifth and eighth amino acid, was at least four-to-five-fold less active than peptide 204–212. This indicates that lysine 208 (position 5 of peptide 204–212) and aspartic acid 211 (position 8) must be present in order to maintain full effectiveness. This fact is reinforced by the presence of these two amino acids, in the same position and spaced by two hydrophobic residues, in both AF-1 and AF-2.[5] The possibility also that valine 209 (position 6) and phenylalanine 210 (position 7) may have a role in the achievement of the full activity cannot be completely ruled out from these data, although AF-2 has leucine in position 7.[5] Leucine 203 (position 3) is different in AF sequences where there is a methionine. Moreover, in this position alanine or norleucine also may be present without loss of activity.[9]

In summary, in this study some of the possible structural requirements for the LC5 derived nonapeptide using two *in vitro* models were investigated. The data obtained indicate that biological activity, fully retained by the seven amino acid fragment, is due to the core region 208–212 where lysine 208 and aspartic acid 211 may play a pivotal role. Since the anti-inflammatory activity originally ascribed to these peptides[5,6] has now been confirmed[7,15] and extended,[16,17] definition of the key amino acids required for biological activity may serve for designing non-peptidergic molecules endowed with this inhibitory effect on the acute inflammatory process.

**Table 2.** Comparison between peptide 204–212 of LC5 and its analogues

| | | Amino acid sequence | | | | | | | | | $IC_{30}$ | |
|---|---|---|---|---|---|---|---|---|---|---|---|---|
| Region | Protein | 1 | 2 | 3 | 4 | 5 | 6 | 7 | 8 | 9 | RSS | WP |
| 204–212 | LC5 | Ser- | His- | Leu- | Arg- | Lys- | Val- | Phe- | Asp- | Lys | 1.00 | 1.00 |
| 231–239 | LC1 | Pro | Gln | * | * | Arg | * | * | Gln | * | 4.50 | 2.50 |
| 223–231 | LC2 | Pro | * | * | Gln | * | * | * | * | Arg | 0.40 | 1.05 |

* Indicates identity with the top peptide sequence. LC, lipocortin; RSS, rat stomach strip model; WP, washed platelet aggregation. $IC_{30}$ values have been compared by taking as 1.00 those of LC5 peptide (10 μM and 400 μM for RSS and WP, respectively).

### References

1. Flower RJ. Lipocortin and the mechanism of action of glucocorticoids. *Br J Pharmacol* 1988; **94:** 987–1015.
2. Cirino G, Peers SH, Flower RJ, Browning JL, Pepinsky RB. Human recombinant lipocortin 1 has acute local anti-inflammatory properties in the rat paw oedema test. *Proc Natl Acad Sci USA* 1989; **86:** 3428–3442.
3. Parente L, Becherucci C, Perretti M, *et al.* Are the lipocortins the second messengers of the anti-inflammatory action of glucorticoids? In: Melli M, Parente L, eds. *Cytokines and Lipocortins in Inflammation and Differentiation.* New York: Wiley-Liss Inc, 1990; 55–68.
4. Errasfa M, Russo-Marie F. A purified lipocortin shares the anti-inflammatory effect of glucocorticosteroids *in vivo* in mice. *Br J Pharmacol* 1989; **97:** 1051–1058.
5. Miele L, Cordella-Miele E, Facchiano A, Mukherjee AB. Novel anti-inflammatory peptides from the region of highest similarity between uteroglobin and lipocortin I. *Nature* 1988; **335:** 726–730.
6. Perretti M, Becherucci C, Mugridge KG, Solito E, Silvestri S, Parente L. A novel anti-inflammatory peptide from human lipocortin 5. *Br J Pharmacol* 1991; **103:** 1327–1332.
7. Douglas GJ, Flower RJ, Parente L, Perretti M. Peptide 204–212 of lipocortin 5 inhibits the generation of a prostacyclin-like factor from rat aorta preparations *in vitro. Prostaglandins* 1992; **44:** 381–388.
8. Vostal JG, Mukherjee AB, Miele L, Shulman NR. Novel peptides from a region of local homology between uteroglobin and lipocortin-1 inhibit platelet aggregation and secretion. *Biochem Biophys Res Commun* 1989; **165:** 27–36.
9. Tetta C, Camussi G, Bussolino F, Herrick-Davis K, Baglioni C. Inhibition of the synthesis of platelet-activating factor by anti-inflammatory peptides (antiflammins). *J Pharmacol Exp Ther* 1991; **257:** 616–620.
10. Vargas JR, Radomski M, Moncada S. The use of prostacyclin in the separation from plasma and washing of human platelets. *Prostaglandins* 1982; **23:** 929–945.
11. Pepinsky RB, Tizard R, Mattaliano RJ, *et al.* Five distinct calcium and phospholipid binding proteins share homology with lipocortin I. *J Biol Chem* 1988; **263:** 10799–10811.
12. Funakoshi T, Abe M, Sakata M, Shoji S, Kubota Y. The functional site of placental anticoagulant protein: essential histidine residue of placental anticoagulant protein. *Biochem Biophys Res Commun* 1990; **168:** 125–134.
13. Funakoshi T, Abe M, Sakata M, *et al.* Novel anticoagulant peptides from the functional site of human placental anticoagulant protein. *Biochemistry Intern* 1991; **24:** 173–180.
14. Barton GJ, Newman RH, Freemont PS, Crumpton MJ. Amino acid sequence of the annexin super-gene family of proteins. *Eur J Biochem* 1991; **198:** 749–760.
15. Lloret S, Moreno JJ. *In vitro* and *in vivo* effects of the anti-inflammatory peptides, antiflammins. *Biochem Pharmacol* 1992; **44:** 1437–1441.
16. Chan CC, Ni M, Miele L, *et al.* Effects of antiflammins on endotoxin-induced uveitis in rats. *Arch Ophthalmol* 1991; **109:** 278–281.
17. Cabre' F, Moreno JJ, Carabaza A, Ortega E, Mauleon D, Carganico G. Antiflammins. Anti-inflammatory activity and effect on human phospholipase $A_2$. *Biochem Pharmacol* 1992; **44:** 519–525.

**Received 14 December 1992; accepted in revised form 1 February 1993**
